# Metabolic Reprogramming and Longevity of Tissue-Resident Memory T Cells

**DOI:** 10.3389/fimmu.2018.01347

**Published:** 2018-06-18

**Authors:** Youdong Pan, Thomas S. Kupper

**Affiliations:** Department of Dermatology, Brigham and Women’s Hospital, Harvard Medical School, Boston, MA, United States

**Keywords:** metabolism, longevity, TRM cells, viral vaccines, cancer immunotherapy

## Abstract

Tissue-resident memory T cells (T_RM_) persist in peripheral tissues for long periods of time in the absence of antigenic stimulation. Upon re-encounter with cognate antigen, T_RM_ trigger an immediate immune response at the local tissue microenvironment and provide the first line of host defense. T_RM_ have been reported to play significant roles in host antimicrobial infection, cancer immunotherapy, and pathogenesis of a number of human autoimmune diseases, such as psoriasis, vitiligo, and atopic dermatitis. T_RM_ display a distinct gene transcriptome with unique gene expression profiles related to cellular metabolism that is different from naive T cells (T_N_), central memory T cells (T_CM_), and effector memory T cells (T_EM_). Skin CD8^+^ T_RM_ upregulate expression of genes associated with lipid uptake and metabolism and utilize mitochondria fatty acid β-oxidation to support their long-term survival (longevity) and function. In this review, we will summarize the recent progresses in the metabolic programming of T_RM_ and will also discuss the potential to target the unique metabolic pathways of T_RM_ to treat T_RM_-mediated diseases.

Memory T cells mediate immunosurveillance and protect the host through rapid recall responses upon re-exposure to previously encountered pathogens (1). In addition to the two previously identified circulating memory T cells, central memory T cells (T_CM_), and effector memory T cells (T_EM_), a new subtype of memory T cells—tissue-resident memory T cells (T_RM_)—has been identified and characterized (2–4). Unlike T_CM_ and T_EM_ that circulate within blood, T_RM_ reside and remain within epithelial barrier tissues for long periods of time without trafficking back into lymph or blood (5). Upon antigen re-exposure, T_RM_ trigger an immediate immune response and provide the first line of protection against the antigen/pathogen they are specific for (4, 6–11). In addition, T_RM_ create a general antiviral microenvironment at the local tissue site and provide cross-protection against antigenically unrelated pathogens (7, 9). Activation of T_RM_ alters tissue-wide gene expression profiles, induces B cell and circulating memory T cell recruitment through IFN-γ-dependent vascular cell adhesion molecule 1 upregulation, and leads to maturation of local dendritic cells and activation of natural killer cells. These activities support the idea that T_RM_ function as a bridge between the adaptive and innate immune system (7, 9). As many viruses show tissue tropism, T_RM_ also provide protective immune responses for the tissue through which it was previously encountered. T_RM_ specific for HSV are in skin (12–14), T_RM_ specific for rotavirus are in gut (6, 15), and T_RM_ specific for influenza are in lung (16–18). Collectively, we propose that sensitization of relatively small numbers of T_RM_ may lead to an amplified signal to more abundant elements of the innate immune system and trigger an organ-wide antiviral state. The placing of adaptive immune memory cells at the body’s interfaces with the environment, and moreover those specific for a given pathogen, speaks to the elegance of adaptive immune memory.

Upon cognate recognition of antigen *via* T-cell receptor, naive T cells (T_N_) undergo extensive clonal expansion and differentiate into several T cell subtypes, including effector T cells (T_eff_) for immediate pathogen elimination and memory T cells for long-term protection (19). Recent studies showed that T cell activation and differentiation are accompanied with and regulated tightly by metabolic reprogramming, presumably to provide the divergent energetic and functional needs for their development, maintenance, and function (20–23). T_N_ primarily depend on glucose catabolism and oxidative phosphorylation (OXPHOS) to derive energy to support the maintenance of their relatively quiescent state. T_eff_ reprogram their metabolic state to anabolism to enable rapid cell division and cytokine production (24). T_eff_ increase glucose acquisition from blood through upregulating gene expression of glucose transporter-1 (Glut 1) and conduct glycolysis (converting glucose into pyruvate with the production of two molecules of ATP) to meet their energy demand (25). Although glycolysis is less efficient in generating ATP compared to OXPHOS, it is faster and thus rapidly accommodates the increased demand for the energy and biomass formation of T_eff_. Unlike T_N_ and T_eff_, T_CM_ utilize endogenously synthesized fatty acids and OXPHOS to support their long-time survival (longevity) and function (26–28). T_CM_ maintain substantial mitochondrial spare respiratory capacity and display increased mitochondrial mass, thus providing metabolic advantage and equipping them for both longevity and the ability of rapid recall upon antigen re-challenge (26). O’Sullivan et al. showed that rather than importing extracellular fatty acids, T_CM_ utilize endogenous fatty acid synthesis and subsequently conduct mitochondrial fatty acid oxidation (FAO) and OXPHOS for their differentiation and maintenance (27). T_CM_ take up extracellular glucose from blood to synthesize fatty acids in the endoplasmic reticulum, a process dependent on lysosomal acid lipase, which is critical in hydrolyzing cholesteryl esters and triglycerides within LDL particles into free cholesterol and free fatty acids (FFAs) (29, 30). Cui et al. additionally showed that interleukin-7, a cytokine critical for T_CM_ differentiation and survival, induced glycerol transport and triacylglycerol synthesis *via* enhanced gene expression of glycerol channel aquaporin 9, thus providing substrates for mitochondria FAO (28). However, compared to the well-defined metabolic reprogramming of circulating memory T cells, the metabolic programs utilized by T_RM_ to dictate their fate differentiation and sustain their longevity and function, are only beginning to be understood.

## T_RM_ Metabolism in Skin

**Figure 1:**
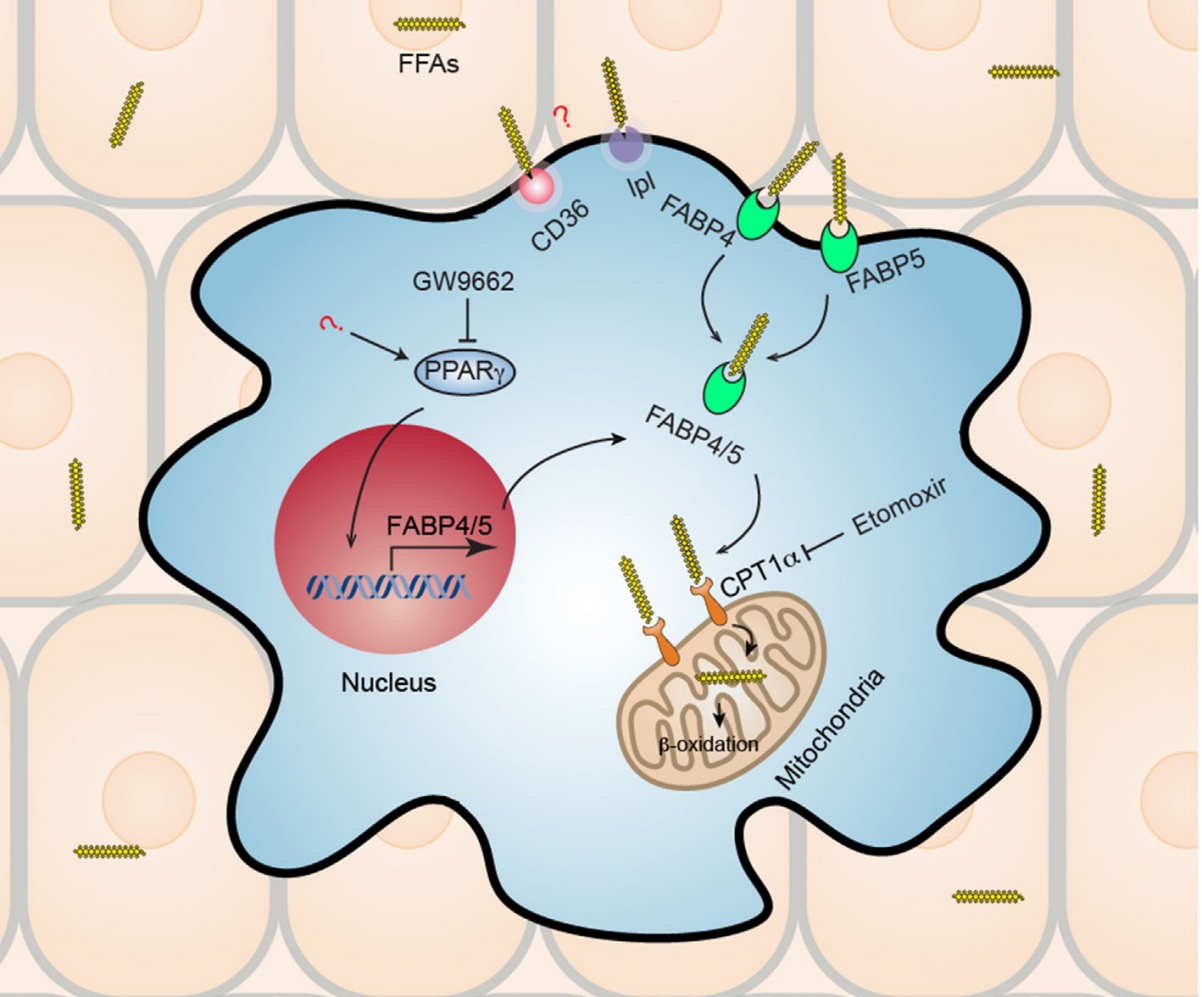
Metabolic reprogramming of skin CD8^+^ tissue-resident memory T cells (T_RM_). Skin CD8^+^ T_RM_ depend on increased uptake of exogenous fatty acid and mitochondrial β-oxidation for their long-term survival (longevity) and function. Skin CD8^+^ T_RM_ upregulate gene expression of transcription factor peroxisome proliferator-activated receptor gamma (PPAR-γ) and its downstream molecules fatty acid binding proteins 4 and 5 (Fabp4/5), which accounts for the increased uptake of free fatty acids (FFAs) from surrounding tissue environment. Subsequently, Skin CD8^+^ T_RM_ utilize mitochondrial fatty acid β-oxidation to generate ATP to support their survival and function. Skin CD8^+^ T_RM_ loss of Fabp4/5 is more prone to cell apoptosis, deficient in long-term survival, and could not protect host efficiently upon viral re-challenge. Treatment with either PPAR-γ inhibitor (GW9662) or with fatty acid mitochondrial β-oxidation inhibitor (etomoxir), results in impaired long-term maintenance of CD8^+^ T_RM_ in skin. In addition, the roles of CD36 and lipoprotein lipase (lpl), both of which are also upregulated in skin CD8^+^ T_RM_ and are involved in lipid metabolism, remain unknown and await to be elucidated by future studies.

Skin, as the primary interface between the body and outer environment, provides a first line of defense against microbial pathogens, physical damage, and chemical insults. In addition to the role of barrier maintenance and sensing, skin functions as a hotbed of immunological activity (31). It has been shown that healthy skin of an adult human being contains about twice T cells as many as are present in the entire blood volume (1 × 10^6^ T cells/cm^2^ and an estimated 2 × 10^10^ T cells in the entire skin surface) (32, 33). T cells contained in human skin are all CD45RO^+^ memory T cells, co-express skin-homing addressin cutaneous lymphocyte-associated antigen and the chemokine receptor CCR4, and more than half of human skin T cells are resident under resting conditions and do not re-circulate (T_RM_) (34). A recent study revealed that pathogenic T cell clones persist in “healed” psoriatic lesions as T_RM_ after complete remission using TNFα blocker (35). Studies on vitiligo showed that vitiligo perilesional skin is enriched with a population of CD8^+^ T_RM_ expressing both CD69 and CD103, in both stable and active disease stages (36, 37). Residing in a nutrient-restricted (particularly glucose) but lipid-rich environments (38, 39), the mechanisms by which skin T_RM_ sustain their longevity and function remained elusive. Using a well-established model of generating CD8^+^ T_RM_ in skin after cutaneous immunization with Vaccinia virus, we showed that skin CD8^+^ T_RM_ adapt to utilize lipid metabolism of exogenous FFAs internalized from the surrounding microenvironment to support both their longevity and protective function (Figure 1) (40). CD8^+^ T_RM_ develop a transcriptional program that features marked overexpression of molecules facilitating exogenous FFAs acquisition and metabolism. Specifically, fatty acid binding proteins 4 and 5 (Fabp4/5), CD36, and lipoprotein lipase (lpl) were in the top 35 most highly overexpressed genes in T_RM_, as compared to T_N_, T_CM_, and T_EM_. Fabp’s are conventionally thought to function as intracellular chaperones for FFAs, shuttling FFAs from cytoplasm to mitochondria for β-oxidation (41). CD36 is a lipid scavenger receptor that binds to and internalizes FFAs and other lipids (42), and lpl is a lipoprotein lipase that cleaves triglycerides to yield a FFA and diacylglycerol (43). This collection of overexpressed genes involved in lipid uptake and metabolism suggested a special relationship between T_RM_ and lipid metabolism. Further study showed that skin CD8^+^ T_RM_ upregulated the gene expression of Fabp4/5 in a peroxisome proliferator-activated receptor gamma (PPAR-γ)-dependent manner. When incubated under the presence of exogenous fluorescently conjugated FFAs, skin CD8^+^ T_RM_ internalized extracellular FFAs much more efficiently compared to other counterparts. Addition of exogenous FFAs induced a significantly higher basal and FCCP-stimulated maximal oxygen-consumption rate in skin CD8^+^ T_RM_, which could be blocked by pretreatment with etomoxir, an irreversible inhibitor of mitochondrial carnitine palmitoyltransferase 1, an enzyme that is the rate limiting step for mitochondrial fatty acid β-oxidation and ATP generation (44). Skin CD8^+^ T_RM_ rendered unable to metabolize exogenous FFAs through mitochondrial β-oxidation, whether through deficiency of Fabp4/5 or pretreatment with etomoxir, cannot persist in skin. By contrast, T_CM_ generated from Fabp4^−/−^Fabp5^−/−^ mice in parallel have no survival disadvantage. Referring to functionality, skin CD8^+^ T_RM_ deficient in Fabp4/5 were inferior in clearing viral infection and insufficient to protect host against lethal viral re-challenge. Consistent with data from mice, CD8^+^ T_RM_ from human skin tissue display higher level of Fabp4/5 expression and internalize more exogenous FFAs compared to other human counterparts, indicating that acquisition of exogenous FFAs for metabolism might represent a conserved feature of T_RM_ across species. Given the dependence of skin CD8^+^ T_RM_ on lipid metabolism and the increasingly uncovered roles of T_RM_ in skin autoimmune diseases such as psoriasis and vitiligo, it is tempting to speculate a novel and promising treatment strategy for skin immune disorders by blocking critical lipid metabolic pathways in T_RM_. Still, much remains to be elucidated further for the mechanism of T_RM_ metabolic maintenance and survival. The precise roles of gene upregulation of PPAR-γ in skin CD8^+^ T_RM_ and the contribution of other lipid metabolism-related genes to the survival and function of skin CD8^+^ T_RM_, such as CD36 and lpl, both of which were also upregulated as part of the T_RM_ transcriptional program, remain to be investigated. Overall, a detailed signaling pathway of T_RM_ metabolism, as well as the cross talk among skin tissue microenvironment, T_RM_ metabolism and effector function, will be of great interest and may facilitate the development of efficient treatment strategy for T_RM_-mediated skin diseases.

## T_RM_ Metabolism in Tumor Microenvironment (TME)

Solid tumors are infiltrated by heterogeneous immune cell types that work in a coordinated fashion to effect antitumor immunity (45). The presence and abundance of tumor-infiltrating lymphocytes (TILs) in tumors is associated with better clinical outcomes after tumor immunotherapy (46–49). TILs differ from their blood counterparts both in terms of upregulated gene expression of immune checkpoint molecules (PD-1, LAG3, TIGIT, and CTLA-4) and reduced effector functions (tumor immunosuppression) (50). Phenotypic analysis of TILs from melanoma revealed that nearly 60% of CD8^+^ T cells and 50% of CD4^+^ T cells are CD45RO^+^CD69^+^CCR7^–^, characteristic of a T_RM_ phenotype (51). Melanoma antigen-specific T_RM_ cells resided predominantly in melanocyte-depleted hair follicles and mediate durable immunity to melanoma (36). Other studies showed that the number of T_RM_ within tumors associates with cytotoxic T cell responses and correlates with a better overall survival in lung cancer, head and neck cancer, and breast cancer (52–54). Local T_RM_ induced *via* immunization through mucosal vaccine inhibited tumor growth (52, 55). Thus, a TIL T_RM_ phenotype is considered as a new surrogate biomarker for the efficacy of cancer vaccines, and development of vaccine strategies designed to generate T_RM_ against tumor cells has attracted great interest as a potentially significant therapeutic application.

Inside tumors, both tumor cells and TILs compete for the oxygen and nutrients supplied *via* infiltrating blood vessels. Rapidly growing tumor cells utilize more glucose and glutamine to conduct glycolysis, resulting in a TME of hypoxia and glucose deprivation for TILs (56, 57). This leads to enhanced expression of immune checkpoint molecules and loss of effector functions in TILs, a process that results in T cell exhaustion (51). On the other hand, TILs conduct metabolic reprogramming to adapt to the metabolic constrains within the TME and sustain their maintenance and antitumor function. Using mouse melanoma models, Ying et al. reported that under short-term hypoxia and hypoglycemia, CD8^+^ T cells decreased transcripts for genes important in glycolysis while increasing transcripts of PPAR-α and downstream molecules involved in FA uptake and mitochondrial FA catabolism (58). Enhanced FA uptake and increased expression of Cpt1a were observed in vaccine-induced CD8^+^ TILs from late-stage tumors, implying the increasing dependence of TILs on fatty acid metabolism for energy maintenance. Promoting fatty acid catabolism with fenofibrate, a PPAR-α agonist, markedly improves the capacity of CD8^+^ TILs to delay tumor growth. This enhancement synergizes with PD-1 blockade to efficiently enhance the efficacy of melanoma immunotherapy. With regard to human tumor biology, the same study reported that TILs isolated from human melanoma metastases show evidence of enhanced FA catabolism, which could be fueled by increased level of FAs within tumor intestinal fluid (58). Collectively, these data suggest that TILs in the TME engage in metabolic reprogramming to utilize FAO for their survival and function. The mechanism by which the TME influences TILs metabolic reprogramming, as well as the nutrient source of FAs for fatty acid catabolism, remains to be further investigated. Also, further studies are required to elucidate the mechanisms by which TILs reprogram their metabolism to cope with the TME and how this metabolic switch affect their survival and antitumor function. Since a growing body of studies support the idea that enhancing already present immune responses against tumors leads to considerably long-lasting tumor remissions and delayed tumor metastasis, a better understanding of TIL T_RM_’s metabolic switch and how to manipulate this process to increase their maintenance and antitumor effector function, may increase the efficacy and improve the outcome of tumor immunotherapy.

## T_RM_ Metabolism in White Adipose Tissue (WAT)

White adipose tissue is a storage depot for fat and an endocrine organ that secretes adipokines to regulate whole-body energy homeostasis and metabolism (59). It connects body barrier surfaces and the internal organs, thereby forming a bridge between tissues that are constantly challenged with surrounding microbes and the inner sterile environments. WAT constitutively regulates glucose and lipid homeostasis by sorting and releasing FFAs *via* lipolysis for usage by other organs (60). Previous work has shed light on cross talks between WAT and immune system in a series of metabolic disorders and inflammatory diseases (61–63). WAT infiltrating lymphocytes are predominantly localized within organized structures referred to as fat-associated lymphoid clusters or milky spots (in the omentum), which can rapidly expand in response to local inflammatory cues (64, 65). Recent studies by Han et al. reveal the residency and occupancy of T_RM_ in WAT and their contribution to immune surveillance and long-term protective memory responses to infection (66). WAT functions as a major hub for adaptive immune memory T cells, predominantly T_RM_. These adipose T_RM_ express a well-established T_RM_ cell surface marker (CD69) and do not equilibrate between the adipose tissue of conjoined naïve and previously infected mice, confirming the residency of these cells. Transplantation of adipose tissue from previously infected mice was sufficient to protect uninfected mice from lethal pathogen challenge, whereas depletion of T cells abrogated this protective effect, indicating a functional protective role of adipose T_RM_ in systemic pathogen challenge. Following gene expression analysis revealed that adipose T_RM_ cells upregulated genes involved in effector functionalities and lipid metabolism. When incubated *ex vivo* with fluorescently labeled long chain fatty acid palmitate (Bodipy FL C16), adipose T_RM_ cells displayed high rates of lipid uptake and mitochondrial respiration compared to their counterparts from spleen and small intestine lamina propria (siLP), while no difference could be observed in FFA uptake between siLP T_RM_ and spleen T_EM_. These data suggest that T_RM_ in WAT might also utilize fatty acid metabolism for their survival and function. To what extent do adipose T_RM_ depend on fatty acid metabolism and the contribution of fatty acid metabolism to their longevity and function remain to be evaluated further. The same study showed that induction of WAT memory responses results in the remodeling of WAT physiology (66). Thus, it would be interesting to investigate the cross-regulation between adipocytes and T_RM_ metabolism within WAT, as well as how to manipulate the regulation of pathways to increase host protection or treat individuals with obesity and metabolic disorders.

## Targeting T_RM_ Metabolic Pathways to Treat Associated Autoimmune Diseases

Targeted therapies are increasingly successful at inducing temporary and partial remissions in organ-specific immune mediated autoinflammatory diseases, but it remains nearly impossible to induce durable remission or cure (6). These autoimmune disorders, including diseases of skin (psoriasis, vitiligo, graft vs host disease), GI tract (Crohn’s disease, ulcerative colitis), lung (asthma), joint (rheumatoid arthritis, spondyloarthropathies), CNS (multiple sclerosis), and endocrine system (Type I diabetes), are increasing in incidence and prevalence. Over the past decade, a line of investigation central to the understanding of diseases pathogenesis leads to the discovery of T_RM_. Increasing evidences from various experimental models and clinical data support a theory that these autoimmune diseases are driven, at least partially, by inappropriate and chronic activation of pathogenic T_RM_ (6). This provides a plausible explanation for the T cell pathogenesis of these diseases and their organ specificity, something that prior explanations of pathogenesis could not adequately explain. This also provides an explanation for the chronicity of these diseases, as T_RM_ are nearly impossible to dislodge from their tissue sites of residence once established. Currently, in clinic these diseases of regional immune hyperactivation (*via* T_RM_) are usually being treated with systemic immunomodulation and immunosuppression. After successful therapy is withdrawn, T_RM_ remain *in situ* and can become reactivated by pathogenic stimuli, thus resulting in disease relapse. Therefore, a therapy that could not only suppress the activity of pathogenic T_RM_ but also dislodge them from their tissue niches has the potential to induce remissions that are much longer and ideally indefinite. The uniqueness of T_RM_ in their dependence on lipid metabolism of FFAs from the external environment makes it a previously unappreciated “Achilles Heel,” and one that could be exploited therapeutically. Indeed, administration *in vivo* with a pharmacologic mitochondrial β-oxidation, trimetazidine [blocks the long chain 3-ketoacyl CoA thiolase activity (67)], decreased the survival and maintenance of T_RM_ in skin (40). Thus, the likelihood exists that pharmacologic approach targeting the lipid metabolic pathway in T_RM_ could reduce, and theoretically eliminate, the pathogenic T_RM_ that are causative in autoinflammatory disorders of multiple tissues.

## Conclusion and Future Perspectives

It has recently become clear that control of metabolism and the adaptive immune system are tightly linked (21, 22, 68). Nutrient availability and cellular metabolism closely regulate the differentiation, survival, and function of immune cells (23). T_RM_ are not simply memory T cells residing in an unexpected location; rather, they are a specific group of memory T cells with unique lineage (40, 69–71). As revealed from gene transcriptional profiling, T_RM_ display a quite distinct transcriptome from those of T_CM_ and T_EM_, both of which were more similar to that of T_N_ (69, 70, 72). Recent findings have shed light on the role of cellular metabolism in regulating differentiation and memory formation of T_CM_ (26–28). However, it remains unknown how cellular metabolism controls T_RM_ fate decision. Moreover, the focus of previous studies on T_RM_ metabolism is primarily on CD8^+^ T_RM_, and little is known about the metabolic reprogramming of CD4^+^ T_RM_ and their roles in CD4^+^ T_RM_ differentiation, survival, and function. In addition, attributed to the restricted nutrient availability at specialized tissue sites, more studies will be required to elucidate the metabolic pathways of T_RM_ at other tissue sites such as lung, intestine, and brain. Finally given that generation of long-lived T_RM_ are a goal of efficient vaccination, and considering the dual role of T_RM_ in tumor and autoimmune tissue disorders, a more detailed understanding of the unique metabolic programs intrinsic to T_RM_, and how these programs might be manipulated to enhance or decrease T_RM_ longevity and function, will be a subject of future study with high clinical relevance and therapeutic significance.

## Author Contributions

YP drafted and edited the manuscript; TK edited and approved the final version of the manuscript.

## Conflict of Interest Statement

The authors declare that the research was conducted in the absence of any commercial or financial relationships that could be construed as a potential conflict of interest.
